# Long Term Exposure to Polyphenols of Artichoke (*Cynara scolymus* L.) Exerts Induction of Senescence Driven Growth Arrest in the MDA-MB231 Human Breast Cancer Cell Line

**DOI:** 10.1155/2015/363827

**Published:** 2015-06-09

**Authors:** Anna Maria Mileo, Donato Di Venere, Claudia Abbruzzese, Stefania Miccadei

**Affiliations:** ^1^Regina Elena National Cancer Institute, Via Elio Chianesi 53, 00144 Rome, Italy; ^2^CNR, Institute of Sciences of Food Production (ISPA), Via Amendola 122/O, 70126 Bari, Italy

## Abstract

Polyphenolic extracts from the edible part of artichoke (*Cynara scolymus* L.) have been shown to be potential chemopreventive and anticancer dietary compounds. High doses of polyphenolic extracts (AEs) induce apoptosis and decrease the invasive potential of the human breast cancer cell line, MDA-MB231. However, the molecular mechanism underlying AEs antiproliferative effects is not completely understood. We demonstrate that chronic and low doses of AEs treatment at sublethal concentrations suppress human breast cancer cell growth via a caspases-independent mechanism. Furthermore, AEs exposure induces a significant increase of senescence-associated *β*-galactosidase (SA-*β*-gal) staining and upregulation of tumour suppressor genes, p16^INK4a^ and p21^Cip1/Waf1^ in MDA-MB231 cells. AEs treatment leads to epigenetic alterations in cancer cells, modulating DNA hypomethylation and lysine acetylation levels in total proteins. Cell growth arrest correlates with increased reactive oxygen species (ROS) production in AEs treated breast cancer cells. Inhibition of ROS generation by N-acetylcysteine (NAC) attenuates the antiproliferative effect. These findings demonstrate that chronic AEs treatment inhibits breast cancer cell growth via the induction of premature senescence through epigenetic and ROS-mediated mechanisms. Our results suggest that artichoke polyphenols could be a promising dietary tool either in cancer chemoprevention or/and in cancer treatment as a nonconventional, adjuvant therapy.

## 1. Introduction

Cancer is one of the main causes of death worldwide, claiming over six million people each year [1]. According to current estimates, cancer is 30–40% preventable over time with appropriate nutrition, regular physical activity, and avoidance of obesity [2].

Chemoprevention is a promising strategy which uses natural dietary compounds and/or synthetic substances to block, inhibit, reverse, or delay the process of carcinogenesis. Important preventive mechanisms include suppression of cell proliferation and apoptosis and modulation of epigenetic processes [3–6].

Many epidemiological studies suggest that diets particularly rich in fruits and vegetables have cancer preventive properties [7–9]. The beneficial effect of diets is attributable, at least in part, to polyphenols which have antitumour activities both in animal models and in humans [8–14]. Furthermore, several clinical trials testing the efficacy of natural compounds are underway, http://www.clinicaltrials.gov/; some of them are already concluded and demonstrate the chemopreventive activity of dietary polyphenols [15–17].

The current growing interest for dietary plants has led to a renewed attention for artichoke because of its high polyphenolic content. In the edible part, it mainly consists of hydroxycinnamic derivatives, in particular chlorogenic and dicaffeoylquinic acids. Our studies of bioavailability demonstrate that ferulic acid is one of the metabolites present in human plasma after an artichoke meal [18]. Preclinical reports indicate that ferulic acid has antiproliferative and chemopreventive activities* in vitro* and* in vivo* [19–21] and proposed the potential use of ferulic acid as an adjuvant agent during chemo- and/or radiotherapy [22, 23].

Our previous findings indicate that polyphenolic artichoke extracts (AEs) protected hepatocytes from oxidative stress and exhibited cancer chemopreventive properties, in part, by triggering apoptosis on human hepatoma cells Hep G2 [24] and on the human breast cancer cell line, MDA-MB231 [25].

Despite the growing scientific results regarding chemopreventive activities of natural dietary compounds [8], the cellular mechanisms underlying antitumour property of polyphenols are yet to be elucidated.

Cellular senescence, a state of cell cycle arrest, can be considered a relevant mechanism of tumour suppression [26–28]. Furthermore, emerging evidence has demonstrated that therapy-induced senescence is a critical mechanism through which many anticancer agents inhibit tumour progression [29–31]. Importantly, therapy-induced senescence can be achieved in administering agents at low doses. This approach can significantly reduce the side effects of conventional anticancer therapy, thus improving the quality of life for cancer patients [29, 30]. Innovative senescence therapies will be developed through improved knowledge of the molecular pathways controlling permanent growth arrest by specifically screening for senescence effectors.

Scientific evidence from* in vitro* studies indicates that the cancer prevention activity involved modulation of epigenetic processes. Epigenetics is defined as heritable changes in gene expression that are not accompanied by alterations in DNA sequence [32]. The main epigenetic processes are DNA methylation, histone modifications, and chromatin remodeling. Aberrant patterns of gene expression are key features of cancer and both genetic and epigenetic abnormalities are implicated in this molecular deregulation. In contrast to genetic modifications, epigenetic alterations are potentially reversible and strategies targeting the epigenome have been proposed for both cancer prevention and therapeutics [33].

Induction of premature senescence and modulation of epigenetic processes have been identified as relevant anticancer features of dietary polyphenolic compounds [34]. There are both* in vitro* and* in vivo *data showing that several bioactive food components can interfere with DNA methylation and histone modifications and affect the expression of genes involved in the carcinogenic process [35]. In particular, phenolic acids, including chlorogenic acid, have been shown to affect DNA methylation [36].

Since the induction of senescence requires moderate concentrations of anticancer agents and produces almost no side effects [37], we sought to investigate whether a low dose and chronic treatment of AEs could inhibit the growth of breast cancer cells through the induction of premature senescence. In the present study, we demonstrate that a moderate and chronic treatment of AEs causes a significant increase in senescence-associated *β*-galactosidase (SA-*β*-gal) detection and upregulation of p21^Cip1/Waf1^ and p16^INK4a^ protein expression in MDA-MB231 cells. Such a premature senescence is via ROS-mediated DNA damage and is associated with the modulation of epigenetic machinery. Altogether, these results highlight a significant contribution of senescence induction to AEs anticancer effects.

## 2. Materials and Methods 

### 2.1. Artichoke Extract Preparation

The edible part (head) of fresh artichoke (*Cynara scolymus* L. cv Violetto di Provenza) buds was used for extract preparation and the analysis of polyphenols contained in the extracts was performed by HPLC as previously described [25].

### 2.2. Cell Lines and Cultured Conditions

Cell lines were maintained in a humidified incubator with 5% CO_2_ and 95% air at 37°C. HCT 116 cells, human colon carcinoma cell line, MDA-MB231, oestrogen receptor-negative breast cancer cells, HEY cells, human ovarian cell line, and K-562 cells, human erythromyeloblastoid leukaemia cell line (kindly supplied by Dr. Maurizio Fanciulli, Dr. Paola Nisticò, and Dr. Maria Giulia Rizzo, Regina Elena National Cancer Institute Rome) were grown in RPMI medium (Invitrogen Life Technologies, Milan, Italy) supplemented with 10% FBS, 10 IU/mL penicillin, and 10 *μ*g/mL streptomycin. GTL-16 cells, human gastric carcinoma cell line, DU 145 cells, human prostate carcinoma cell line, A549 cells, human lung carcinoma cell line, M14 cells, human melanoma cell line, U-373 MG cells, human astrocytic cell line, and Saos-2 cells, human osteosarcoma cell line (kindly supplied by Dr. Giovanni Blandino, Regina Elena National Cancer Institute, and Dr. Antonella Farsetti, CNR Rome, purchased from American Type Culture Collection, Rockville, MD, USA) were grown in D-MEM supplemented with 10% FBS, 10 IU/mL penicillin, and 10 *μ*g/mL streptomycin.

### 2.3. Reagents

Artichoke extracts were dissolved in PBS and 0.1% dimethylsulfoxide (Me_2_SO, Sigma-Aldrich, Milan, Italy). Paclitaxel (Ptx, Sigma-Aldrich) was dissolved in PBS. 5-Bromo-4-chloro-3-indolyl-*β*-d-galactopyranoside (X-gal) was purchased from IBI Scientific (Peosta IA, USA) and used as previously described [38]. N-Acetyl-cysteine (NAC, Sigma-Aldrich) was dissolved in PBS. Dihydroethidium (DHE, Molecular Probes-Invitrogen UK) was dissolved in Me_2_SO.

### 2.4. Cytotoxicity and Cell Proliferation Assays

Human cancer cells were seeded at density of 2.0 × 10^5^ in 6-well plates (Sarstedt, Numbrecht, Germany) in triplicate and cultured for 24 h before adding either the vehicle (0.1% Me_2_SO) or AEs. Cytotoxicity experiments were assessed in serum-free medium and cells were treated with AEs (200, 400, 600, and 800 *μ*M) for 24 h.

For the cell proliferation experiment, MDA-MB231 were seeded at a density of 5 × 10^3^ in 6-well plates in triplicate. After 24 h the cells were exposed to AEs (10 and 30 *μ*M) for 10 days. To determine the role of ROS in AEs-mediated senescence, cells were pretreated with 10 mM NAC for 2 h and then exposed to AEs (10 and 30 *μ*M) for 10 days.

Cells were harvested by trypsinization, stained with 0.4% trypan blue (Sigma-Aldrich), and counted using a haemocytometer.

### 2.5. Senescence-Associated *β*-Galactosidase (SA-*β*-gal) Assay

MDA-MB231 cells were seeded at a density of 5 × 10^3^ in 6-well plates in triplicate and cultured for 24 h before adding either the vehicle or AEs (10 and 30 *μ*M). After 10 days of a chronic treatment, with every 48 h renewal of medium and treatment, cultured cells were washed in PBS, fixed in 2% formaldehyde/0.2% glutaraldehyde for 15 minutes at room temperature. Cells were then washed and incubated with fresh SA-*β*-gal staining solution containing 1 mg/mL X-gal, 40 mM citric acid/sodium phosphate (pH 6.0), 5 mM potassium ferrocyanide, 5 mM potassium ferricyanide, 150 mM NaCl, and 2 mM MgCl_2_ for 16–18 h at 37°C. Blue-stained senescent cells were counted using a light microscopy (Olympus IX71-Olympus America, Center Valley, PA, USA) and the images were captured by digital camera (Olympus-Camedia C-5060).

### 2.6. Western-Blot Analysis

To prepare the whole-cell extract, cells were washed with PBS and suspended in ice cold RIPA lysis buffer (50 mM Tris-HCl pH 8, 1 mM EDTA, 150 mM NaCl, 1% NP40, 1% sodium deoxycholate, 0.1% SDS) with protease inhibitors. After 30 minutes of mixing at 4°C, the mixture was centrifuged (10,000 ×g) for 10 minutes and the supernatants were collected as whole-cell extracts. The protein content was determined with a protein assay reagent (Bio-Rad, Milan, Italy) using bovine serum albumin as a standard. An equal protein content of total lysates (25 *μ*g) from the control and from AEs treated samples was resolved on 10% SDS-PAGE with molecular weight markers (Bio-Rad). Proteins were then transferred to the PVDF membrane (EMD Millipore Billerica, MA, USA) and reacted with anti-p16^INK4a^ and anti-p21^Cip1/Waf1^ antibodies (Santa Cruz Biotechnology, CA, USA), acetylated-lysine polyclonal antibody (Cell Signalling Technology Inc., Danvers, MA, USA), and anti-actin antibody (Calbiochem-EMD Millipore Billerica, MA, USA) for protein normalization. The protein bands were revealed by chemiluminescence using an ECL detection kit (Amersham Bioscience, Cologno Monzese, Milan, Italy). Autoradiograms were quantified with ImageJ 1.43 software (National Institutes of Health, Bethesda, MD, USA).

### 2.7. Dot Blot Analysis of DNA 5-Methylcytosine and 5-mC Immunostaining

MDA-MB231 cells were seeded at a density of 2.5 × 10^3^ in 6-well plates in triplicate and cultured for 24 h before adding either the vehicle or various concentrations of AEs (2.5, 5, 10, and 30 *μ*M) for 10 days and then harvested. Genomic DNA was isolated using the Wizard DNA purification kit (Promega, Madison, WI, USA) according to the manufacturer's instructions, transferred onto positively charged Hybond N+ membranes (Amersham Pharmacia Biotech, Piscataway, NJ, USA) using dot filtration apparatus and fixed by baking the membrane for 30 minutes at 80°C. After baking, membranes were incubated with 5 *μ*g/mL of 5-methylcytosine (5-mC) antibody (Calbiochem, San Diego, CA, USA) followed by incubation with a horseradish peroxidase-conjugated secondary antibody. Membranes were then treated with chemiluminescence detection reagents and exposed to Kodak autoradiograph films. The intensity of each dot was quantified with ImageJ 1.43 software.

For the immunostaining analysis, cells (1 × 10^5^) were cytospun using a Shandon Cytospin 3 (Thermo Scientific, Waltham, MA, USA) at 1000 rpm for 10 minutes and then processed for 5-mC immunostaining. Cells were permeabilized with 0.4% triton X-100 in PBS for 20 minutes. Cells were washed with PBS for 10 minutes and then blocked for 30 minutes with 3% preimmune goat serum in PBS. After 20 minutes of incubation with 3% H_2_O_2_ in order to quench endogenous peroxidase the cells were washed with PBS and incubated with 5-mC specific antibody (1 : 500 vol/vol) for 2 h. Cells were sequentially incubated with biotinylated prediluted antibody, horseradish peroxidase-conjugated streptavidin (UltraTek HRP, ScyTek Laboratories Inc, Logan, UT, USA) and AEC substrate (AEC substrate kit, ScyTek Laboratories Inc.) and counterstained with haematoxylin.

### 2.8. Intracellular ROS Detection

MDA-MB231 cells were treated with either the vehicle or AEs (10 and 30 *μ*M). After 10 days, the oxidation-sensitive fluorescent probe DHE was used to assess the production of cytosolic superoxide anions. Briefly, at the end of exposure, cells were incubated with 5 *μ*M DHE for 40 minutes at 37°C in the dark and then rinsed twice with PBS. The cell-permeant DHE entered the cells, was oxidized by superoxide anions to form ethidium (ETH) which binds to DNA, and produced the fluorescent ETH-DNA. The fluorescent signals were obtained exciting the cultured cells at *λ*_ex_ 300 nm and *λ*_em_ 610 nm. Cells were visualized and counted by fluorescence microscope (Olympus IX71-Olympus America).

The role of ROS on AEs-treated cell proliferation was evaluated using the antioxidant NAC. Cells were preincubated with 10 mM NAC for 2 h and then exposed to AEs. After 10 days, cells were harvested by trypsinization, stained with 0.4% trypan blue, and counted using a haemocytometer.

### 2.9. Statistical Analysis

Statistical analyses were performed by Student's *t*-test using GraphPad 5.1 software. For all statistical tests, a two-tailed *p* value <0.05 was considered significant. All data reported were verified at least in three independent experiments and expressed as mean ± SD.

## 3. Results

### 3.1. Phenolic Composition of Artichoke Extracts

The artichoke extracts were found to contain monocaffeoylquinic acids (MCQA), dicaffeoylquinic acids (DCQA), and small amounts of a luteolin and an apigenin glycoside. The main phenolic components of the AEs found were chlorogenic acid and two dicaffeoylquinic acids (3,5-DCQA and 1,5-DCQA) at a ratio of about 1 : 1 : 1. The concentrations of chlorogenic acid, 3,5-DCQA, and 1,5-DCQA, determined by HPLC, as previously described [25], were found to be 725 ± 70, 738 ± 58, and 632 ± 48 mgL^−1^, respectively.

### 3.2. Effects of AEs on Human Cancer Cell Lines Viability

We have previously reported that AEs exhibited cancer chemopreventive activities on a human hepatoma cell line, Hep G2 [24], and on a human breast cancer cell line, MDA-MB231 [25]. To investigate whether the antiproliferative activity of AEs could be extended to other tumours, we describe the effect of AEs on 10 cancer cell lines derived from various human tissues, as shown in Table 1. This panel provides a way of presenting the cellular sensitivity or resistance at three levels of effect. After 24 h, 800 *μ*M AEs caused about 50% of death in prostate and melanoma cells; more than 50% of toxicity was detected in breast, ovary, lung, brain, bone, and leukaemia cells. Less than 50% of dead cells were observed in colon and gastric cancers. The more resistant cancer cells, such as gastric and colon, showed sensitivity to 1200 *μ*M AEs (unpublished results). These data indicated that high doses of AEs are cytotoxic for all tested cancer cell lines without any effect on untreated counterparts.

### 3.3. Low Doses of AEs Inhibit Breast Cancer Cell Growth via a Caspases-Independent Mechanism

**Figure 1:**
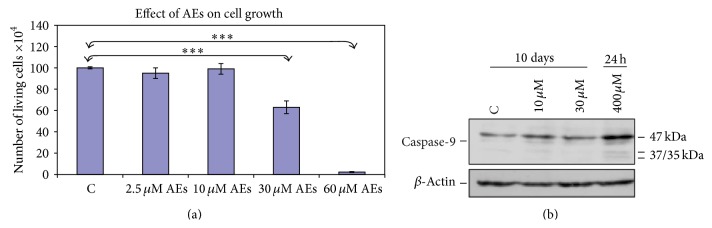
Low doses of AEs suppress breast cancer cell growth via a caspases-independent mechanism. (a) Cell growth inhibition by AEs. MDA-MB231 cells were treated with increasing concentrations of AEs (from 2.5 to 60 *μ*M) for 10 days. The results are the mean ± SD of at least three independent experiments. Significant statistical differences are indicated by asterisks: ^*∗∗∗*^ *p* < 0.0001. (b) Effects of AEs on caspases pathway. The cells were treated with low doses of AEs (10 and 30 *μ*M) for 10 days or with a high concentration of AEs (400 *μ*M) for 24 h, as a positive control. Whole cell lysates (25 *μ*g/lane) were tested for activation and cleavage of caspase-9. *β*-Actin was used as a protein loading control.

We have previously demonstrated [25] that high concentrations of AEs (from 200 to 800 *μ*M for 24 h) are able to activate an apoptotic program in MDA-MB231 to halt tumour progression. Since bioactive compounds concentrations required to induce apoptosis in tumour cell lines might not be reachable in target tissues, we asked whether AEs chronically administered at low and sublethal doses can affect the growth of tumour cells as well. To this purpose, we focused on MDA-MB231, which provide the scientific rationale for testing AEs as an antitumour agent against invasive and hormone resistant breast cancer phenotype. After 10-day treatment (from 2.5 *μ*M to 60 *μ*M) direct cell count assay indicated that the cellular growth was significantly inhibited by chronic exposure to low doses of AEs (Figure 1(a)). Treatments up to 30 *μ*M resulted in a relevant inhibition of cell proliferation in viable cells (about 90%); the highest concentration (60 *μ*M) induced a dramatic growth arrest and was slightly cytotoxic (about 70% viability). Based on these data, we focused on two noncytotoxic concentrations of AEs, namely 10 and 30 *μ*M, that modulate cell growth. Furthermore, we investigated whether these treatments caused a reduction in the number of living cells through the activation of caspases pathways. As shown in Figure 1(b), in these experimental conditions protein expression analysis revealed that caspase-9 was not activated. Conversely, high concentration of AEs (400 *μ*M), used as positive control [25], triggers a significant activation/cleavage of caspase-9 after 24 h treatment. In addition, long term exposure to low concentrations of AEs did not activate caspase-8 (unpublished results). Altogether our results strongly suggested that low doses of AEs inhibit breast cancer cell growth via a caspases-independent mechanism.

### 3.4. AEs Induce Premature Senescence in Breast Cancer Cells

**Figure 2:**
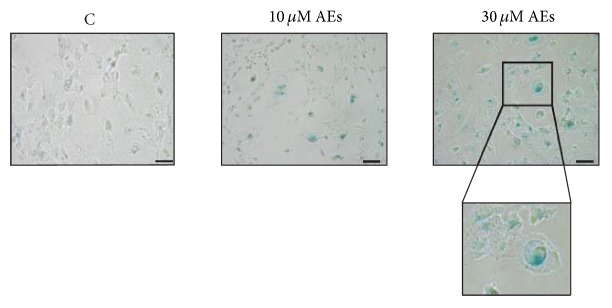
Chronic treatment of AEs induces cell senescence. MDA-MB231 cells were treated with low doses of AEs (10 and 30 *μ*M) for 10 days and then analyzed for the SA-*β*-gal, senescence-associated *β*-galactosidase activity. The image shown is representative of at least three independent experiments. Scale bar: 50 *μ*m. The senescent cells versus total cells were counted in random fields under an inverted microscope (20x) and the following data are the mean ± SD: C = 7.7 ± 0.5, 10 *μ*M AEs = 31 ± 5.6, *p* = 0.0044^*∗∗*^, 30 *μ*M AEs = 51 ± 6.6, *p* = 0.0005^*∗∗∗*^. Significant statistical differences are indicated by asterisks. Boxed area, regarding blue cells with a typical senescent flattened and enlarged morphology, is magnified (2x).

We investigated whether the cell growth arrest, in response to low doses of AEs, was caused by the induction of cellular senescence as demonstrated for chronic treatment of several polyphenols in cancer cells [39–42]. The detection of *β*-galactosidase positive cells reflects an increase in lysosomal mass in aging cells and it is widely regarded as a marker for senescence [43]. MDA-MB231 cells incubated without AEs showed no detectable SA-*β*-gal activity, whereas cells treated with 10 and 30 *μ*M AEs revealed a marked X-gal staining in a dose-dependent manner after 10 days, as depicted in Figure 2. Most of the SA-*β*-gal positive cells showed an enlarged and flattened morphology with increased volume and granularity that were consistent with cellular senescence status, as shown in magnification area of Figure 2.

**Figure 3:**
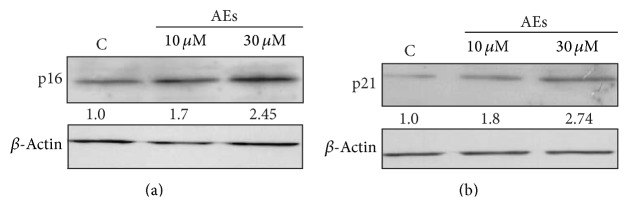
Chronic treatment of AEs induces increased p16^INK4a^ and p21^Cip1/Waf1^ expression. MDA-MB231 cells were treated with low doses of AEs (10 and 30 *μ*M) for 10 days. Whole cell lysates were tested for (a) p16^INK4a^ and (b) p21^Cip1/Waf1^ protein expression. Immunoblots are representative of at least three independent experiments. Intensities of electrophoretic bands relative to the immunoblot shown were quantified by densitometry and the values are reported.

To further investigate this antiproliferative mechanism, we examined the expression levels of p21^Cip1/Waf1^ and p16^INK4a^, two pivotal cell cycle regulators involved in cellular senescence [44, 45]. Western blotting data demonstrated that the expression levels of p21^Cip1/Waf1^ and p16^INK4a^ were significantly increased in a dose-depending manner in AEs-treated cells (Figures 3(a) and 3(b)). Optical density measurements were performed using ImageJ software to obtain quantitative values for the protein expressions. Altogether, these findings suggested that low doses and chronic exposure to AEs induced senescence in MDA-MB231 breast cancer cells.

### 3.5. Effect of AEs on Global Methylation Level in Breast Cancer Cells

**Figure 4:**
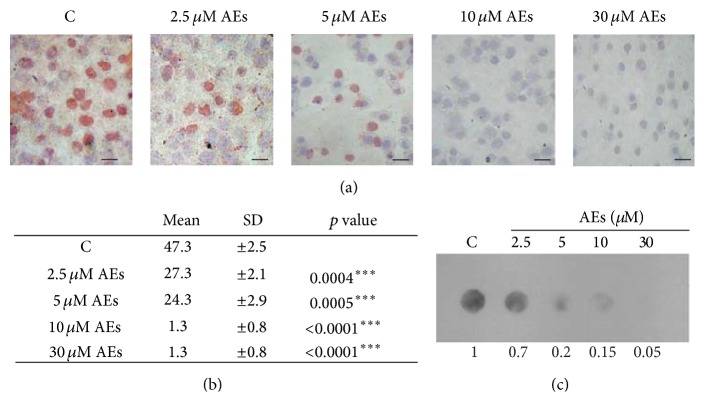
Effect of AEs on global DNA methylation levels in MDA-MB231 cells. Low doses of AEs (2.5–30 *μ*M) for 10 days treatment decreased levels of 5-mC, 5-methylcytosine, in a dose-dependent manner. 5-mC specific antibody was used for (a) and (b) cytostaining and (c) for dot blot analysis on genomic DNA. (a) The image shown is representative of at least three independent experiments. Scale bar: 100 *μ*m. (b) The 5-methylcytosine positive cells versus total cells were counted by using an inverted microscope (20x) and the results are reported as mean ± SD. (c) The intensity of individual DNA dots shown was quantified by densitometry.

DNA hypermethylation is a major epigenetic modification that leads to silencing of tumour suppressor genes which may contribute to cancer development and progression [46]. Recent investigations suggest that some dietary phytochemicals may prevent cancer by modulating epigenetic processes [35, 47]. To examine whether chronic treatment has epigenetic effects on the DNA methylation level, cells were exposed to increasing concentrations of AEs, as shown in Figure 4. After 10 days, cells were harvested for immunocytostaining of DNA methylation using an antibody specific to 5-methylcytosine (5-mC). Treatment of AEs resulted in a reduced number of 5-mC-positive cells in a dose-dependent manner compared to untreated cells (Figures 4(a) and 4(b)).

To further verify the effect of AEs on DNA methylation, genomic DNA was isolated from cells and analysed by dot blot assay using anti-5mC antibody. As shown in Figure 4(c), AEs treatment of MDA-MB231 cells significantly decreased the DNA global methylation level as quantified by densitometric values. These results demonstrated that AEs have a relevant effect on regulation of DNA methylation machinery.

### 3.6. Effect of AEs on Acetylation of Total Proteins on Human Breast Cancer Cells

**Figure 5:**
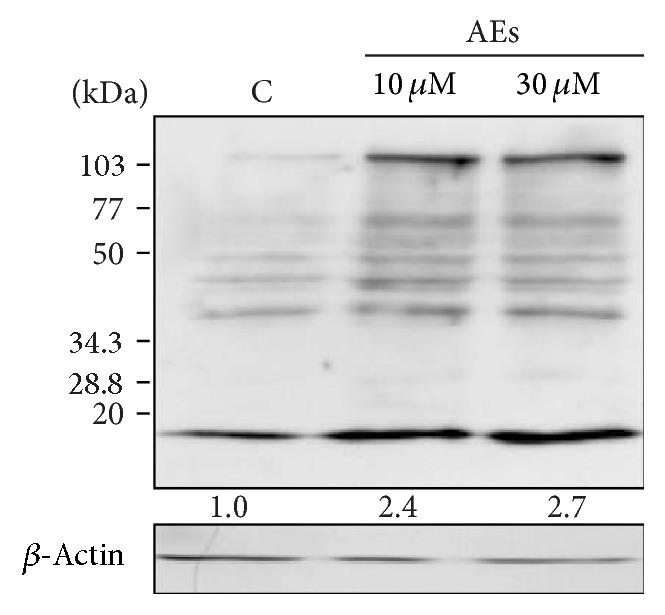
Effect of AEs on lysine acetylation of total proteins in MDA-MB231 cells. Cells were treated with low doses of AEs for 10 days. Cell lysates were analyzed for lysine acetylation of total proteins. Immunoblot is representative of at least three experiments. The intensities of electrophoretic bands relative to the immunoblot shown were quantified by densitometry.

Protein acetylation of lysine residues is an important reversible modification controlling cellular protein expression [48]. We investigated the effect of AEs treatment on acetylation of total proteins in breast cancer cells. MDA-MB231 cells were cultured for 24 h before adding either the vehicle or various concentrations of AEs (10 and 30 *μ*M) for 10 days and then harvested. As shown in Figure 5, the level of protein acetylation is markedly increased in treated cells as indicated by reported densitometric values. These results provide evidence that long term exposure to low concentrations of AEs is associated with increased level of lysines acetylation of total proteins.

### 3.7. AEs Treatment Increases ROS Production in Breast Cancer Cells

**Figure 6:**
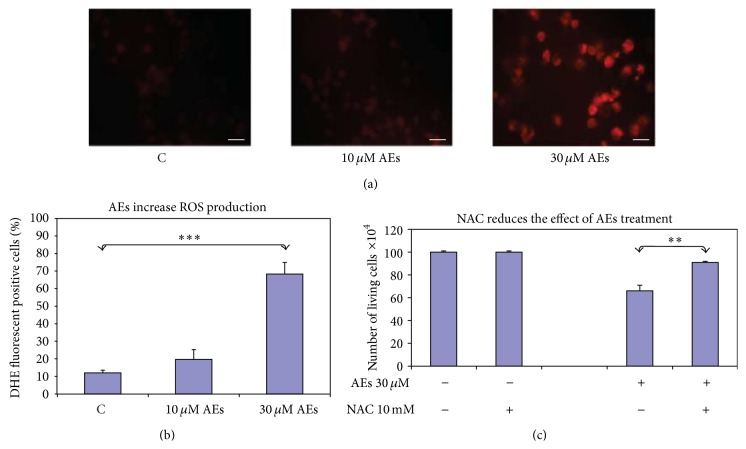
ROS production in MDA-MB231 cells treated with AEs. (a) The presence of ROS was detected by DHE fluorescent staining after 10 days treatment. Scale bar: 50 *μ*m, original magnification 20x. (b) Red fluorescent-stained cells versus total cells were counted using an inverted fluorescence microscope. The results are the mean ± SD of at least three independent experiments. Significant statistical differences are indicated by asterisks: ^*∗∗*^ *p* = 0.0012. (c) NAC reduced the antiproliferative effect of AEs (30 *μ*M for 10 days). The results are the mean ± SD of at least three independent experiments. Significant statistical differences are indicated by asterisks: ^*∗∗*^ *p* = 0.0012.

Since reactive oxygen species (ROS) are well-known inducers of cellular senescence, we tested whether AEs increase oxidative stress in breast cancer cells. To accomplish this, cells were incubated with DHE which is an indicator of the presence of superoxide anion, key radical in ROS generation. As shown in Figures 6(a) and 6(b) AEs-treated cells, according to the increased number of bright red fluorescent cells, showed enhanced level of superoxide anions compared to control cells.

To determine the role of ROS in AEs-induced growth arrest, we sought to examine whether inhibition of ROS production by antioxidant NAC has any impact on senescence in breast cancer cells. As shown in Figure 6(c), the presence of NAC significantly reduced the antiproliferative effect of 30 *μ*M AEs for long term exposure. Furthermore, NAC treatment decreased percentage of SA-*β*-gal positive cells (unpublished results). According to data in literature regarding the natural products modulation of ROS expression in senescence [39, 41, 49], our findings strongly support the hypothesis that an oxidative pathway is involved in AEs-induced growth arrest in breast cancer cells.

## 4. Discussion

In this report, we provide evidence demonstrating that low doses and chronic AEs-treatments exert anticancer activity through induction of premature senescence in MDA-MB231, a triple negative and highly aggressive breast cancer cell line. Experimental data demonstrate that moderate doses of AEs inhibit the growth of breast cancer cells via a caspases-independent mechanism.

Several reports point to a crucial physiological role for cellular senescence in fighting tumorigenesis. Cellular aging is a state of cell cycle arrest induced at the end of the cellular life span or in response to agents causing oxidative stress and DNA damage. Moreover, senescence is regarded as an important mechanism either for fighting premalignant tumours and/or for inducing tumour regression [2] since it limits the proliferative capacity of cells.

Increasing evidence has demonstrated that many phytochemicals exert anticancer and chemopreventive activities through the induction of senescence growth arrest [39–42].

Beside cell cycle inhibitors, p21^Cip1/Waf1^ and p16^INK4a^ are identified as senescence markers [44, 45].

Both p21^Cip1/Waf1^ and p16^INK4a^ pathway induction can lead to the inhibition of pRb phosphorylation by inhibiting CDK2/cyclinE and CDK4/cyclinD complex, respectively. Furthermore, p21^Cip1/Waf1^ and p16^INK4a^ are likely to cooperate to keep pRb in a hypophosphorylated form during senescence [50]. According to data regarding the prosenescence property of a derivative of caffeic acid [51], we showed that low doses of AEs containing mono- and dicaffeoylquinic acids induce an increased number of SA-*β*-gal positive and flattened cells with enhanced expression of p21^Cip1/Waf1^ and p16^INK4a^.

High doses of AEs treatment induces cell death [25] in breast cancer as well as in other cell lines derived from various human cancers (Table 1) whereas AEs chronically administered on MDA-MB231 exert antiproliferative activity via induction of a caspases-independent mechanism.

This is a significant finding since the major challenge for anticancer therapeutic strategy leading to apoptosis* in vitro* is that effective concentrations of an antitumour agent should be too high to be reachable* in vivo* [52].

Data in literature are consistent with our results since dietary polyphenols [42, 49, 53, 54] activate apoptotic machinery when used at high doses whereas low level treatments induce senescence in cancer cells.

The senescence program involves epigenetic processes such as DNA methylation, histone modifications, and chromatin remodeling. DNA hypermethylation is a major epigenetic mechanism in the silencing of tumour suppressor genes. Protein lysine acetylation is a posttranslational change that has long been known to play a prominent role in the regulation of gene expression via modulation of chromatin structure. This epigenetic process involves modification of histone and nonhistone proteins through acetyltransferase (HATs) and deacetylase (HDACs) activities. Abnormal DNA methylation and dysregulated histone acetylation are implicated in numerous reported diseases, including cancer [55]. In contrast to genetic modifications, epigenetic deregulation is potentially reversible and therapeutic strategies, targeting the epigenome, have been proposed for both cancer prevention and clinical treatment [56]. Many epigenetic modulators have been used in clinical trials (either completed or terminated) to treat human cancers (http://www.clinicaltrials.gov/). According to results of preclinical studies and clinical trials, epigenetic modulators, administered as monotherapy or in combination with conventional chemo- and radiotherapies, are potentially very useful [57].

Modulation of epigenetic processes has been identified as a relevant anticancer feature of many dietary polyphenolic compounds [33, 34, 58, 59]. There are both* in vitro* data and* in vivo *data showing that several bioactive food components can interfere with DNA methylation and histone modification affecting the expression of gene involved in the carcinogenic process [35]. Epigallocatechin-3-gallate (EGCG), the major polyphenolic constituent of green tea, has been demonstrated to inhibit DNA methyl transferase in several cancer cell lines, including MDA-MB231 cells [60]. This activity was associated with promoter demethylation and reactivation of p16^INK4a^. The positive clinical efficacy of EGCG in the treatment or prevention of chronic lymphocytic leukaemia [16] or prostate cancer [15] has been evaluated. A synergistic mixture of green tea plus capsicum has been demonstrated to have a chemopreventive effect versus different types of cancer in subjects testing positive for ENOX2, (ecto-nicotinamide adenine dinucleotide oxidase disulfide-thiol exchanger 2), which is ideally suited as a target for early diagnosis of cancer as well as for preventive intervention [17]. Curcumin has the potential to treat a wide variety of diseases including cancer [61]. Its epigenetic modulator activity has been a focus in clinical trials (terminated or completed). Phenol derivatives, including quercetin and resveratrol, have been shown to possess epigenetic properties through sirtuin activation [57]. Phenolic acids, including chlorogenic acid, the main component of AEs, have been shown to affect DNA methylation [36].

According to these data, we observed consistent DNA hypomethylation and increased total acetylation protein levels in AEs-treated MDA-MB231 cells.

It has been demonstrated that several anticancer polyphenolic compounds from fruit and vegetables induce tumour cellular growth arrest largely through the generation of ROS [62]. Low doses of resveratrol inhibit cell growth and enhance radiosensitization via the induction of ROS-mediated premature senescence in lung cancer cells [39, 49]. Sin et al. suggest that chronic treatment with 20(S)-ginsenoside Rg3, a compound extracted from ginseng, at a subapoptotic concentration caused senescence-like growth arrest and increased ROS production in human glioma cells [54]. Moreover, bisdemethoxycurcumin, a natural derivative of curcumin, suppresses human breast cancer cell proliferation by inducing oxidative stress senescence [41]. A relevant role of ROS was also demonstrated for the phenethyl isothiocyanate induction of apoptosis and senescence in tumours [53]. Altogether, these findings suggest the crucial role of ROS as effectors of polyphenol-induced prooxidant damage in cancer cells. Accordingly, we show that AEs-induced growth arrest is associated with increased ROS production, suggesting that AEs may induce senescence via an oxidative-mediated damage in breast cancer cells. To confirm this important contribution of ROS, we demonstrate that antioxidant NAC attenuates AEs proliferative effect on MDA-MB231 cells.

It is important to highlight that we have previously shown that AEs have a prooxidant activity on breast cancer cells [25] and an antioxidant effect on normal hepatocyte [24]. Given that aberrant redox system is frequently observed in many tumour cells [63], we hypothesize that AEs may selectively inhibit the growth of tumour cells with little or no toxicity on normal cells based on their differential redox status.

Conventional cancer therapy is traditionally based on the efficacy of cytotoxic treatments even though severe side effects on patients are a clinical relevant problem. An alternative strategy is the induction of cytostatis, which disables the proliferative capacity of cells without inducing cancer cell death. A promising cytostatic approach is prosenescence therapy which may provide a relevant growth inhibitory effect in both early and late stage cancers [29, 64]. Targeted prosenescence therapies may be of remarkable clinical interest since it might minimize toxicity and improve quality of life for cancer patients [29, 30]. Over the last few years, it has been demonstrated that several prosenescence polyphenols and natural compounds could represent a promising novel therapeutic approach for cancer intervention [39, 41, 42, 54, 65]. The suppressive role of senescence in cancer progression has promoted the idea that induced premature cell aging could be an alternative or a complement to conventional anticancer treatments.

In line with this, our present study demonstrates for the first time that AEs may induce ROS accumulation in MDA-MB231 breast cancer cells and modulate the p21^Cip1/Waf1^ and p16^INK4a^ pathways to cause a senescence-mediated tumour suppression. Our study adds a novel aspect of the underlying mechanisms of the anticancer properties of AEs. However, in order to provide a solid basis for evaluating the efficacy in human clinical trials, further studies are required on animal models. In particular, deep pharmacokinetic and metabolic studies of AEs are needed.

In conclusion, our findings propose dietary artichoke polyphenols as a very promising tool either for the management of cancer prevention in healthy, high risk breast cancer women or for the design of innovative, nonconventional, adjuvant therapies in cancer treatment.

## Figures and Tables

**Table 1:** Cytotoxicity of AEs in human cancer cell lines.

	AEs *μ*M									
	NT		200		400		600		800	
	Mean	SD	Mean	SD	Mean	SD	Mean	SD	Mean	SD
HCT 116	84.5	3.7	77.2	6.1	84.0	2.7	79.0	4.0	77.0	4.5
GLT-16	93.5	1.3	89.2	0.9	89.5	4.5	83.2^**^	2.7	69.7^***^	3.6
MDA-MB231	90.0	2.6	83.7	1.5	81.7^*^	1.0	43.2^***^	1.0	16.0^***^	1.4
HEY	91.7	2.2	91.0	3.6	90.0	2.1	73.5^*^	4.9	27.7^***^	1.7
DU 145	92.7	2.3	93.0	2.9	94.0	2.4	80.0^**^	3.4	51.0^***^	2.9
A549	86.5	5.9	86.2	5.5	82.5	11.2	70.0	2.0	28.3^**^	3.5
M14	88.2	5.6	83.0	2.9	77.7	10.9	74.3	2.5	39.6^**^	3.2
U-373 MG	93.7	3.7	95.0	1.4	90.2	6.3	91.5	1.7	16.5^***^	2.0
Saos-2	89.8	2.3	86.5	6.5	85.3	4.6	37.3^***^	6.9	18.3^***^	1.5
K-562	89.7	4.3	15.2^***^	4.3	2.7^***^	1.2	3.7^***^	1.5	1.5^***^	0.6

Ten tumour cell lines derived from various human tissues are treated with increasing concentrations of AEs (from 200 to 800 *μ*M) for 24 h. The results are the mean ± SD of at least three independent experiments. The statistical significance between groups was calculated using Student's *t*-test. Significant differences are indicated by asterisks.

GLT-16: ^**^ *p* = 0.0092, ^***^ *p* = 0.0009.

MDA-MB231: ^*^ *p* = 0.0129, ^***^ *p* < 0.0001.

HEY: ^*^ *p* = 0.0119, ^***^ *p* < 0.0001.

DU 145: ^**^ *p* = 0.0041, ^***^ *p* < 0.0001.

A549: ^**^ *p* = 0.0029.

M14: ^**^ *p* = 0.0023.

U-373 MG: ^***^ *p* = 0.0001.

Saos-2: ^***^ *p* < 0.0002.

K-562: ^***^ *p* < 0.0002.
